# Gene mutation‐based and specific therapies in precision medicine

**DOI:** 10.1111/jcmm.12722

**Published:** 2016-03-15

**Authors:** Xiangdong Wang

**Affiliations:** ^1^ Zhongshan Hospital Biomedical Research Center Fudan University Medical School Fudan University Center for Clinical Bioinformatics Shanghai Institute of Clinical Bioinformatics Shanghai China

**Keywords:** precision medicine, gene mutation, therapy strategy, gene sequencing

## Abstract

Precision medicine has been initiated and gains more and more attention from preclinical and clinical scientists. A number of key elements or critical parts in precision medicine have been described and emphasized to establish a systems understanding of precision medicine. The principle of precision medicine is to treat patients on the basis of genetic alterations after gene mutations are identified, although questions and challenges still remain before clinical application. Therapeutic strategies of precision medicine should be considered according to gene mutation, after biological and functional mechanisms of mutated gene expression or epigenetics, or the correspondent protein, are clearly validated. It is time to explore and develop a strategy to target and correct mutated genes by direct elimination, restoration, correction or repair of mutated sequences/genes. Nevertheless, there are still numerous challenges to integrating widespread genomic testing into individual cancer therapies and into decision making for one or another treatment. There are wide‐ranging and complex issues to be solved before precision medicine becomes clinical reality. Thus, the precision medicine can be considered as an extension and part of clinical and translational medicine, a new alternative of clinical therapies and strategies, and have an important impact on disease cures and patient prognoses.

Precision medicine is a concept to treat disease according to gene abnormality, a strategy to cure patients by manipulating an alteration of the exact gene, and an initiative to fight the most difficult diseases. For decades, clinicians have dreamt about a concept like precision medicine that is different from both individualized and personalized therapies. Since Barack Obamas’ announcement on the launch of a new Precision Medicine Initiative earlier this year, precision medicine has been gaining popularity and attention. The concept of precision medicine, its main components, and development strategies of the initiative have been further explained where cancer was considered as the first targeting disease 1. While cancer and diabetes are the initial diseases that the Precision Medicine Initiative focuses on, there has also been significant attention on a large number of other diseases worldwide. Although one can certainly use this special term, it is also preferred to use the more common term ‘personalized medicine’. Using ‘precision medicine’ somehow could lead the reader into a perhaps false direction, because finally what is meant in practice resembles the attempts to attack diseases, such as cancer, with a more personalized approach than has been the standard hitherto.

Precision medicine is suggested as a new emerging area and therapeutic strategy to bring unexpected successes and as a new path to improve the treatment and prognosis of patients. We initially described and emphasized five key elements or critical parts in precision medicine and suggested the establishment of a systems understanding of precision medicine 2. For example, the importance of clinical bioinformatics was repeatedly emphasized as one key element to integrate clinical phenotypes and informatics with bioinformatics, computational science, mathematics and systems biology. More accurate and repeatable methodologies for the identification and validation of gene discovery are critical and necessary in the performance of precision medicine. Precision medicine is highly dependent upon new therapeutic strategies, drug discovery and development, and gene‐oriented treatment. The efficacy and application of precision medicine have to be monitored by disease‐specific, mechanism‐based or epigenetics‐dependent biomarkers, and ensured by ‘precision’ regulations 2.

The principle of precision medicine is proposed to treat patients on basis of genetic alterations, after gene mutations are identified. A large number of questions and challenges should be seriously considered to perform precision medicine in clinic. For example, there is a great and urgent need to establish powerful ‘big data’ bases that contain the standardized reference of gene profiles (*e.g*. expression, mutation or epigenetics) in normal populations, since most of published data of gene profiles were concluded in small populations. The wide variety of methodologies to identify and validate disease‐specific alternations of gene profiles results in a lower accuracy of the measurements, which have been questioned. Lander (2015) offered a clear perspective and prospect of precision medicine and defined an important challenge to have rapid innovations and developments of precision medicine and simultaneously ensure patient safety 3. One of the biggest challenges faced is how to identify and validate disease subtype‐, staging‐, severity‐, duration‐ and therapy‐specific targets of gene profiles, since the heterogeneity of gene expression and mutation normally exists among individuals, populations and races. It is also important which mutations are harmless and harmful in a disease, since a large number of harmless gene mutations exist.

When designing therapeutic strategies for precision medicine, biological and functional variations should be carefully considered and their importance should be emphasized. There is currently confusion between strategies to target and correct disease‐specific and dependent gene mutation during therapies. There also remain misunderstandings in how to measure gene mutations as drug‐sensitive parameters. For example, epidermal growth factor receptor (EGFR) is frequently overexpressed in non‐small cell lung cancer (NSCLC), and is associated with cancer cell survival, proliferation, invasion and metastasis by balancing with the hepatocyte growth factor/c‐Met signal 4. The EGFR gene and/or protein expression was suggested to be dependent on histological subtypes and impact survival, rather than prognosis 5. In a meta‐analysis containing about 3000 patients, EGFR overexpression was seen in 39% of adenocarcinoma, 58% in squamous cell carcinoma and 38% in large cell carcinoma, where it was not associated with a poorer prognosis. Based on the information of EGFR gene or protein expression, a number of EGFR inhibitors (EGFR TKIs) were discovered and developed with a special indication for NSCLC. Actually, EGFR inhibitors play reversible or irreversible competitive inhibition roles of the tyrosine kinase domain of EGFR that binds to its adenosine‐5′ triphosphate‐binding site, rather than gene mutation corrections. Somatic activating mutations of the EGFR gene were found to be associated with cancer responses, sensitive or resistant, to therapies for NSCLC, especially EGFR inhibitors, *e.g*. exon 19 deletion mutations and the single‐point substitution mutation L858R in exon 21 are the most frequent in NSCLC. The point mutation T790M EGFR mutation was proposed to be responsible for about 50% of acquired resistance against EGFR inhibitors. Recent clinical study demonstrated that Rociletinib, an EGFR inhibitor, was effective in patients with EGFR‐mutated NSCLC associated with the T790M resistance mutation, demonstrated by the finding that the objective response rate among the 46 patients with T790M‐positive disease was 59% and the rate among the 17 patients with T790M‐negative disease was 29% 6. However, we have to be aware that there is no evidence to show that EGFR inhibitors can interfere with or correct copy number abnormalities or mutations of EGFR gene, and that EGFR inhibitors are designed on the basis of EFGR kinase structure and activities, rather than gene mutations.

The therapeutic strategy of precision medicine should be to identify gene mutations and abnormalities firstly, on which drugs can be designed to ‘correct’ the abnormality of the specific gene. Such strategy requires a number of criteria to be practicable, *e.g*. a powerful gene sequencing in a certain amount of population, a disease‐specific mutation gene, a mechanism‐based validation, and clinically applicable restoration of mutated gene. An outstanding study of such a strategy and selected the adenomatous polyposis coli (APC) tumour suppressor‐mutated gene for restoration in human colorectal cancers 7. The reasons for the selection of APC were 90% of the colorectal tumours contain inactivating APC mutations. Individuals with specific germline mutations in APC invariably develop colon cancer before the age of 35. Adenomatous polyposis coli mutant colorectal cancer accounts for more than 600,000 deaths annually and globally. Adenomatous polyposis coli suppression was suggested to be the critical molecular mechanism for adenomas development in the small intestine and colon and cancer progression in the presence of Kras and p53 mutations. Adenomatous polyposis coli restoration was found to accelerate cancer cell differentiation and sustain regression without relapse by the reestablishment of normal crypt‐villus homoeostasis. Adenomatous polyposis coli restoration can revert colorectal cancer cells to functioning normal cells, even though potent oncogenic insults such as Kras and p53 mutations are present 7. Studies from Daw *et al*. (2015) demonstrate an important potential for the future of therapeutic strategy in precision medicine and provide a new precision medicine strategy, *i.e*. re‐engaging the endogenous tumour suppression mechanisms, for the clinical utility.

There is an important need to repeat and validate such strategies of gene restoration in human cancer and find the applicable solution to translate such strategies into clinical practice. Precision/Personalized cancer medicine is based on increased knowledge of the cancer mutation repertoire and availability of agents that target altered genes or pathways. Given the recent advances in cancer genetics, technology, and therapeutics development, we should furthermore consider and explore the possibilities to develop clinical trials and research frameworks to move future clinical decisions from heuristic to evidence‐based decisions which are based on individual mutation and personalized data.

Therapeutic strategies of precision medicine can be developed mainly according to gene mutations, epigenetics and variations, which are urgently needed to understand and target gene‐specific mechanisms responsible for the development and formation of gene abnormalities, *e.g*. tautomerism, depurination, deamination, slipped strand mispairing, error prone replication by‐pass, errors introduced during DNA repair and induced mutation. It is necessary to identify variations and specificities of structure‐, function‐, fitness‐, inheritance‐based and associated abnormalities. A new therapeutic strategy for human mitochondrial diseases was recently demonstrated by selectively eliminating mitochondrial DNA mutations using mitochondria‐targeted nucleases and preventing their transgenerational transmission 8. Mitochondrial disease is an inherited chronic illness caused by mutations in mitochondrial DNA, resulting in a large number of debilitating physical, developmental and cognitive disabilities with a huge group of clinical symptoms. The specific reduction in mitochondrial genomes in the germline using mitochondria‐targeted restriction endonucleases and transcription activator‐like effector nucleases could prevent those genome transmissions to the next generation. Mutated mitochondrial genomes responsible for human mitochondrial diseases in mouse oocytes were successfully reduced using mitochondria‐targeted nucleases 7. Such approaches can be applied as the new strategy to prevent the transgenerational transmission of human mitochondrial diseases.

In spite of the evidence from gene therapy experiments, it seems not to be so easy that the disease will be cured after a simple replacement of a defective gene or genetic sequence, unless it is monogenetic diseases. From experiments with genetically modified animals with diabetes mellitus, replacing the defective gene and enabling the animals to produce insulin does not cure the diabetes 9. We should realize the possibilities and understand even more the associated problems, such as epigenetics, to make it clear that replacing a genetic code will not automatically cure the underlying disease, despite being the ultimate goal of precision or personalized medicine. The potential financial burden of performing precision medicine needs to be targeted and explored more. Genetic testing and sequencing are still quite expensive, although it may be cheaper in future, and over performed in the population. Such financial burden may become the overload and extra cost for patients and societies. There is still a need for clinical laboratories to have a true and practical guide to perform the gene sequencing at this very moment. Challenges arise with the individual testing of drugs on the basis of genetic information that are not standardized yet.

In addition to precision medicine, other approaches, such as tissue engineering, should be not ignored, but be further explored as therapy modalities 10, 11. Regulation of signalling pathways and non‐coding RNAs including microRNAs and long non‐coding RNAs in the plasticity of tumour stem cells during tumour growth and metastasis can be an alternative of therapies 11. The mesenchymal‐like and epithelial‐like states of circulating stem cells can be targeted to eliminate those lethal seeds of cancers. Stem cells such as neural stem cells and mesenchymal stem cells (MSCs) also can be used to deliver drugs or RNA to the brain through the barrier. Stem cell treatment to deliver drugs to neural tumours is currently in the clinical trial phase. More importantly, MSCs showed pathotropism by migrating to sites of tissue insult. Drug‐engineered MSCs can be available as off‐the‐shelf cells for rapid transplantation across allogeneic barrier 12. With improved understanding of specific somatic mutations or amplifications, single gene tests have an important impact on drug development and cancer treatment. As the spectrum of cancer mutations is extremely diverse in terms of type, number and functional consequences, we need to establish a gigantic knowledge data base to build our therapy decisions on. It is known that mutations are abundant in cancer cells—numbering between thousands and hundreds of thousands per tumour 13. However, most of these mutations in cancer cells do not appear to play a role in cancer progression, but are rather more indicative of the high mutation rate resulting from carcinogens and DNA instability 14.

The importance of gene mutation‐specific and dependent therapeutic strategies and clarification of the ‘precision’ concept in precision medicine should be highly emphasized. A number of strategies in cancer have been suggested as the part of precision medicine, *e.g*. application of a kinase inhibitor with measurement of gene mutations to show which mutation group is more sensitive, or identified gene mutations and then use of the correspondent protein inhibitor 15, 16. It is time to explore and develop a new strategy to target and correct mutated genes, *e.g*. direct elimination, restoration, correction or repair of mutated sequences/genes. Different from inherited diseases, multiple gene mutations and epigenetic alterations are involved in the pathogenesis of cancer or diabetes. Roles of driver gene mutations in mechanism‐based specificity of disease subtypes, stages, severities and responses should be further clarified and validated in a large population. Thus, precision medicine can be considered as an extension and part of clinical and translational medicine, a new alternative of clinical therapies and strategies, and an important impact on disease cures and patient prognoses.

## Conflicts of interest

The authors confirm that there are no conflicts of interest.

## References

[1] Collins FS , Varmus H . A new initiative on precision medicine. N Engl J Med. 2015; 372: 793–5. 25635347 10.1056/NEJMp1500523 PMC5101938

[2] Chen CS , He MY , Zhu YC , *et al* Five critical elements to ensure the precision medicine. Cancer Metastasis Rev. 2015; 34: 313–8. 25920354 10.1007/s10555-015-9555-3

[3] Lander ES . Cutting the Gordian helix ‐ regulating genomic testing in the era of precision medicine. N Engl J Med. 2015; 372: 1185–6. 25689017 10.1056/NEJMp1501964

[4] Wang XD , Li K , Chen H , *et al* Does hepatocyte growth factor/c‐Met signal play synergetic role in lung cancer? J Cell Mol Med. 2010; 14: 833–9. 20178463 10.1111/j.1582-4934.2010.01040.x PMC3823115

[5] Nakamura H , Kawasaki N , Taguchi M , *et al* Survival impact of epidermal growth factor receptor overexpression in patients with non‐small cell lung cancer: a meta‐analysis. Thorax. 2006; 61: 140–5. 16284218 10.1136/thx.2005.042275 PMC2104592

[6] Sequist LV , Soria JC , Goldman JW , *et al* Rociletinib in EGFR‐mutated non‐small‐cell lung cancer. N Engl J Med. 2015; 372: 1700–9. 25923550 10.1056/NEJMoa1413654

[7] Dow LE , O'Rourke KP , Simon J , *et al* Apc restoration promotes cellular differentiation and reestablishes crypt homeostasis in colorectal cancer. Cell. 2015; 161: 1539–52. 26091037 10.1016/j.cell.2015.05.033 PMC4475279

[8] Reddy P , Ocampo A , Suzuki K , *et al* Selective elimination of mitochondrial mutations in the germline by genome editing. Cell. 2015; 161: 459–69. 25910206 10.1016/j.cell.2015.03.051 PMC4505837

[9] Atkinson M . Genetic engineering [internet]. Diapedia. 2014; 0104282147

[10] Horch RE , Beier JP , Kneser U , *et al* Successful human long‐term application of *in situ* bone tissue engineering. J Cell Mol Med. 2014; 18: 1478–85. 24801710 10.1111/jcmm.12296 PMC4124030

[11] Zhu Y , Luo M , Brooks M , *et al* Biological and clinical significance of cancer stem cell plasticity. Clin Transl Med. 2014; 3: 32. 26932376 10.1186/s40169-014-0032-3 PMC4883980

[12] Aleynik A , Gernavage KM , Mourad YS , *et al* Stem cell delivery of therapies for brain disorders. Clin Transl Med. 2014; 3: 24. 25097727 10.1186/2001-1326-3-24 PMC4106911

[13] Stratton MR . Exploring the genomes of cancer cells: progress and promise. Science. 2011; 331: 1553–8. 21436442 10.1126/science.1204040

[14] Pleasance ED , Cheetham RK , Stephens PJ , *et al* A comprehensive catalogue of somatic mutations from a human cancer genome. Nature. 2010; 463: 191–6. 20016485 10.1038/nature08658 PMC3145108

[15] He M , Xia J , Shehab M , *et al* The development of precision medicine in clinical practice. Clin Transl Med. 2015; 4: 69. 26302883 10.1186/s40169-015-0069-y PMC4547974

[16] Abraham E , Marincola FM , Chen Z , *et al* Clinical and translational medicine: integrative and practical science. Clin Transl Med. 2012; 1: 1. 23369263 10.1186/2001-1326-1-1 PMC3552563

